# Mapping the Academic and Global Research Impact of Vagus Nerve Stimulation in Psychiatry: A 25-Year Scientometric Study

**DOI:** 10.7759/cureus.106969

**Published:** 2026-04-13

**Authors:** Ali Imam Awan, Nida Waheed, Eslam Elhossieny, Rida Zahra, Deepan Singh

**Affiliations:** 1 Psychiatry, Maimonides Medical Center, Brooklyn, USA; 2 Medicine, Mayo Hospital, Lahore, PAK; 3 Neurology, Punjab Institute of Neurosciences, Lahore, PAK

**Keywords:** bibliometric analyses, clinical psychiatry, interventional procedures, neuromodulation therapy, vagus nerve stimulator

## Abstract

Vagus nerve stimulation (VNS) has emerged as a significant neuromodulatory intervention for treatment-resistant psychiatric disorders. Despite its expanding clinical applications, a comprehensive assessment of the scientific landscape informing VNS use in psychiatry remains limited. This review maps the global academic research impact of VNS in psychiatry through a 25-year scientometric analysis of the 100 most cited original research articles indexed in Scopus between 2000 and 2025. The analysis examined publication trends, author networks, institutional and geographic output, citation metrics, and thematic concentration. The field demonstrated a 6.21% annual growth rate, with publication peaks in 2005, 2007, and 2020. The selected articles received 4,693 total citations (mean: 82.48), authored by 594 individuals across 35 journals, with the United States dominating both output and citation impact. Treatment-resistant depression emerged as the dominant thematic focus. This review provides a critical academic roadmap for research prioritization, faculty development, and curricular advancement in neuromodulation, with direct relevance to academic psychiatrists, educators, and departmental leaders.

## Introduction and background

Vagus nerve stimulation (VNS) is a therapeutic approach that modifies autonomic nervous system activity to treat refractory psychiatric and neurological conditions. It has emerged as a promising intervention for treatment-resistant depression and has gained attention for its potential role in post-traumatic stress disorder (PTSD), anxiety disorders, and epilepsy-associated psychiatric symptoms [1-3]. VNS is also being explored for inflammation-related psychiatric conditions, reflecting growing interest in its neuroimmune and autonomic mechanisms of action [2].

Psychiatric disorders impose a substantial global burden, with the World Health Organization estimating that nearly one billion individuals are affected worldwide, contributing significantly to disability and socioeconomic impact [4]. Despite advances in pharmacotherapy and psychotherapy, a substantial proportion of patients remain treatment-resistant, highlighting the need for innovative interventions like VNS [5]. The U.S. Food and Drug Administration's approval of VNS for treatment-resistant depression in 2005 marked a pivotal shift in neuromodulation research, catalyzing investigations into its mechanisms and broader applications [6].

Given the increasing volume and complexity of VNS research in psychiatry, there is a need to systematically map its scholarly trajectory and identify key trends, contributors, and knowledge gaps. Bibliometric analysis serves this purpose by quantifying scholarly output, mapping conceptual development, and spotlighting influential and neglected domains [7]. While bibliometric studies have examined neuromodulation techniques such as transcranial magnetic stimulation (TMS) and deep brain stimulation (DBS), as well as psychiatric disorders like depression and schizophrenia, no prior analysis has focused specifically on VNS within psychiatric research [8,9].

This bibliometric investigation serves a dual purpose: to quantitatively map the landscape of VNS research in psychiatry and to inform academic psychiatry leadership, program directors, research mentors, and junior investigators about institutional trends, scholarly impact, and future opportunities. Given the increasing integration of neuromodulation in residency education and clinical innovation, our findings provide a foundational reference for strategic planning in academic psychiatry.

## Review

Search strategy and study selection

A comprehensive bibliometric analysis was conducted to examine the most influential original research articles on VNS in psychiatric disorders. The Scopus database was selected as the primary data source due to its extensive indexing of peer-reviewed literature and its robust bibliometric capabilities. The search was performed in February 2025 following a pre-defined search strategy to ensure reproducibility and accuracy. The search targeted English-language original articles on VNS and psychiatric disorders published between 2000 and 2025. Filters were applied to limit results to peer-reviewed journal articles (DOCTYPE: ar, SRCTYPE: j). Review articles, guidelines, and articles lacking citation data were excluded. The search query included variations of "vagus nerve stimulation" and "psychiatric disorders" using the TITLE-ABS-KEY fields. Two independent reviewers (NW and AIA) screened articles to confirm relevance, and articles without accessible abstracts were cross-verified through institutional repositories or publisher platforms. Disagreements during selection were resolved by consensus. For reproducibility purposes, the final search query was as follows: TITLE-ABS-KEY(("vagus nerve stimulation" OR "vagal nerve stimulation") AND ("psychiatric disorder*" OR "depression" OR "anxiety" OR "PTSD" OR "mental disorder*")) AND DOCTYPE(ar) AND SRCTYPE(j) AND PUBYEAR > 2000 AND PUBYEAR < 2025 AND LANGUAGE(english).

Data extraction and analysis

Data fields extracted from Scopus included citation counts, year of publication, author names, institutional affiliations, journal sources, and author-assigned keywords. Citation-based metrics such as total citations, citations per year, and document age were analyzed using descriptive statistics. Author productivity was evaluated using both raw article counts and fractional authorship contribution, which adjusts for co-authorship by proportionally attributing credit to each listed author. Journal-level impact was assessed using h-index, g-index, and m-index values to capture citation strength and time-adjusted performance. Geographic and institutional contributions were examined by aggregating metadata by country and affiliation, with citation influence reported as total and average citations per entity. Visualization of results, including bar graphs, funnel plots, and sunburst charts, was performed in Microsoft Excel (Microsoft Corporation, Redmond, WA, USA) [10]. All statistical analyses were conducted using R version 4.3.1 (R Foundation for Statistical Computing, Vienna, Austria) with the Bibliometrix package (version 4.2; developed by Massimo Aria and Corrado Cuccurullo, University of Naples Federico II, Naples, Italy) for bibliometric analysis and normalization [11,12].

Overall bibliometric data of the top 100 cited articles

This analysis of the 100 most cited original research articles on VNS in psychiatric disorders, indexed in Scopus between 2000 and 2025, provides critical insights into the structural and collaborative dynamics of this evolving field. These studies, authored by 594 researchers, reflect a highly interdisciplinary and collaborative domain, spanning psychiatry, neuroscience, biomedical engineering, and translational therapeutics. The absence of single-authored publications underscores the complexity of VNS research and the need for cross-disciplinary coordination. Approximately 27% of the articles involved international co-authorship, and the average of 8.7 co-authors per study highlights the collaborative demands of clinical and mechanistic neuromodulation research. A sustained annual publication growth rate of 6.21% demonstrates increasing academic investment in neuromodulation as an evolving adjunct to conventional psychiatric treatment, especially for refractory conditions. Across the dataset, the articles received 4,693 total citations, with an average of 82.5 citations per article and a mean document age of 12.6 years, demonstrating both academic influence and long-term relevance.

Publication trends (2000-2025)

**Figure 1:**
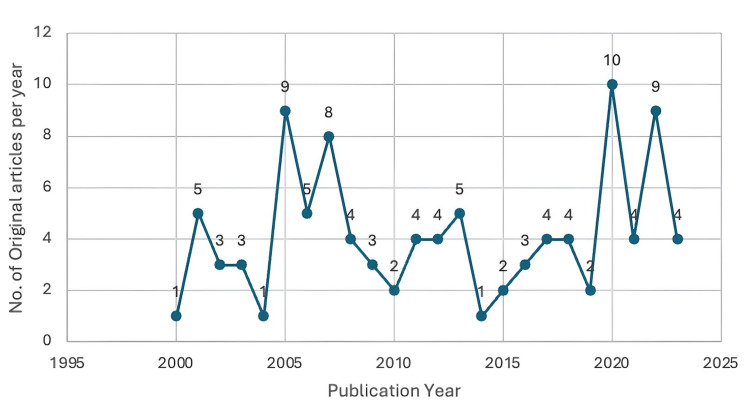
Annual distribution of top-cited VNS articles related to psychiatry (2000–2025) VNS: vagus nerve stimulation This figure has been created by the authors using Microsoft Excel (Microsoft Corporation, Redmond, WA, USA).

The publication trajectory reveals gradual but steady growth in VNS research for psychiatric disorders, with intermittent surges. The years 2005 and 2007 marked early inflection points, each producing eight to nine highly cited articles. A peak followed in 2020 with 10 top-cited publications; this temporal coincidence with the COVID-19 pandemic may speculatively reflect renewed interest in non-invasive, home-based neuromodulation, though no content-based analysis was performed to confirm this association. While output from 2021-2023 stabilized at four articles annually, some years (e.g., 2014, 2019) had minimal presence, potentially due to shifts in research priorities or a lag in citation accrual. Overall, the data demonstrate that VNS research has evolved through early foundational work, periods of intensified activity, and a recent trend toward consistent academic interest, underscoring its growing significance in neuropsychiatric therapeutics. This temporal pattern is illustrated in Figure 1, which presents the annual distribution of publications from 2000 to 2025.

Average citations per year

Citation trajectories further reflect evolving academic engagement. Articles from 2000-2002 garnered the highest average annual citations (more than 20 per year), consistent with foundational contributions to the field. Between 2003 and 2008, citation rates declined modestly before stabilizing post-2010. Modest upticks in 2011, 2015, and 2017 likely correspond to methodologically robust clinical studies. From 2019 onward, a slight rise in citation rate is evident despite limited time for accumulation, indicating growing academic interest in recent VNS research.

Author contributions

**Figure 2:**
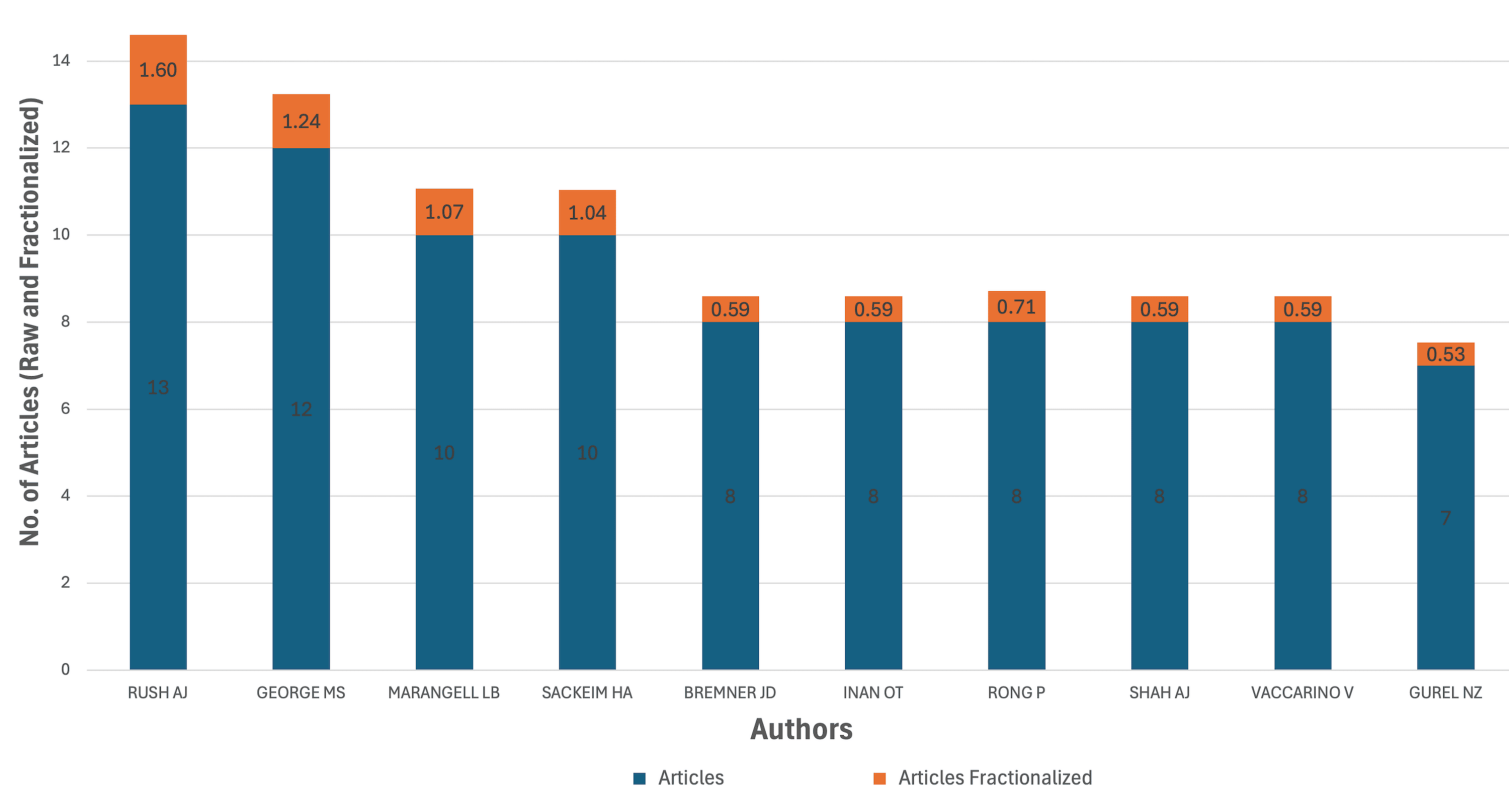
Authorship distribution and fractionalized contributions among top-cited VNS researchers in psychiatry VNS: vagus nerve stimulation This figure has been created by the authors using Microsoft Excel (Microsoft Corporation, Redmond, WA, USA).

A.J. Rush (13 articles) and M.S. George (12 articles) emerged as the most prolific authors, reflecting their leading roles in clinical translation of VNS for affective disorders. L.B. Marangell and H.A. Sackeim (10 each) contributed foundational neuropsychiatric work. Additional frequent contributors, such as J.D. Bremner, O.T. Inan, P. Rong, and A.J. Shah, represent overlapping domains in psychiatric, neural engineering, and translational research. Fractional authorship analysis, which adjusts for co-authorship volume, reaffirmed Rush (score: 1.60) and George (1.24) as central figures, followed by Marangell (1.07) and Sackeim (1.04). Figure 2 presents a visual summary of both raw and fractionalized article counts among the top contributing authors.

Distribution across journals and citation impact

**Figure 3:**
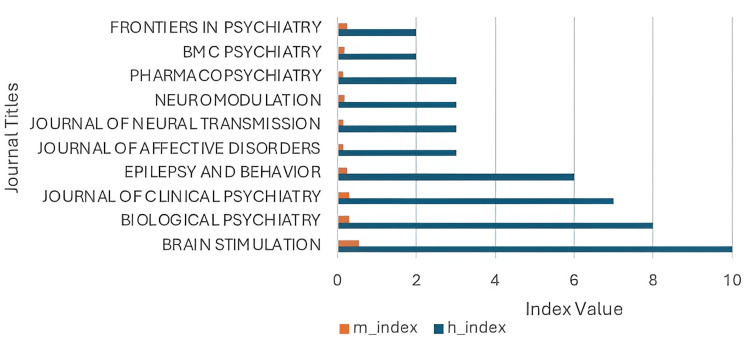
Comparative h-index and M-index of top journals publishing VNS research in psychiatry The h-index indicates citation impact, while the m-index adjusts this impact for years since first publication. VNS: vagus nerve stimulation This figure has been created by the authors using Microsoft Excel (Microsoft Corporation, Redmond, WA, USA).

**Figure 4:**
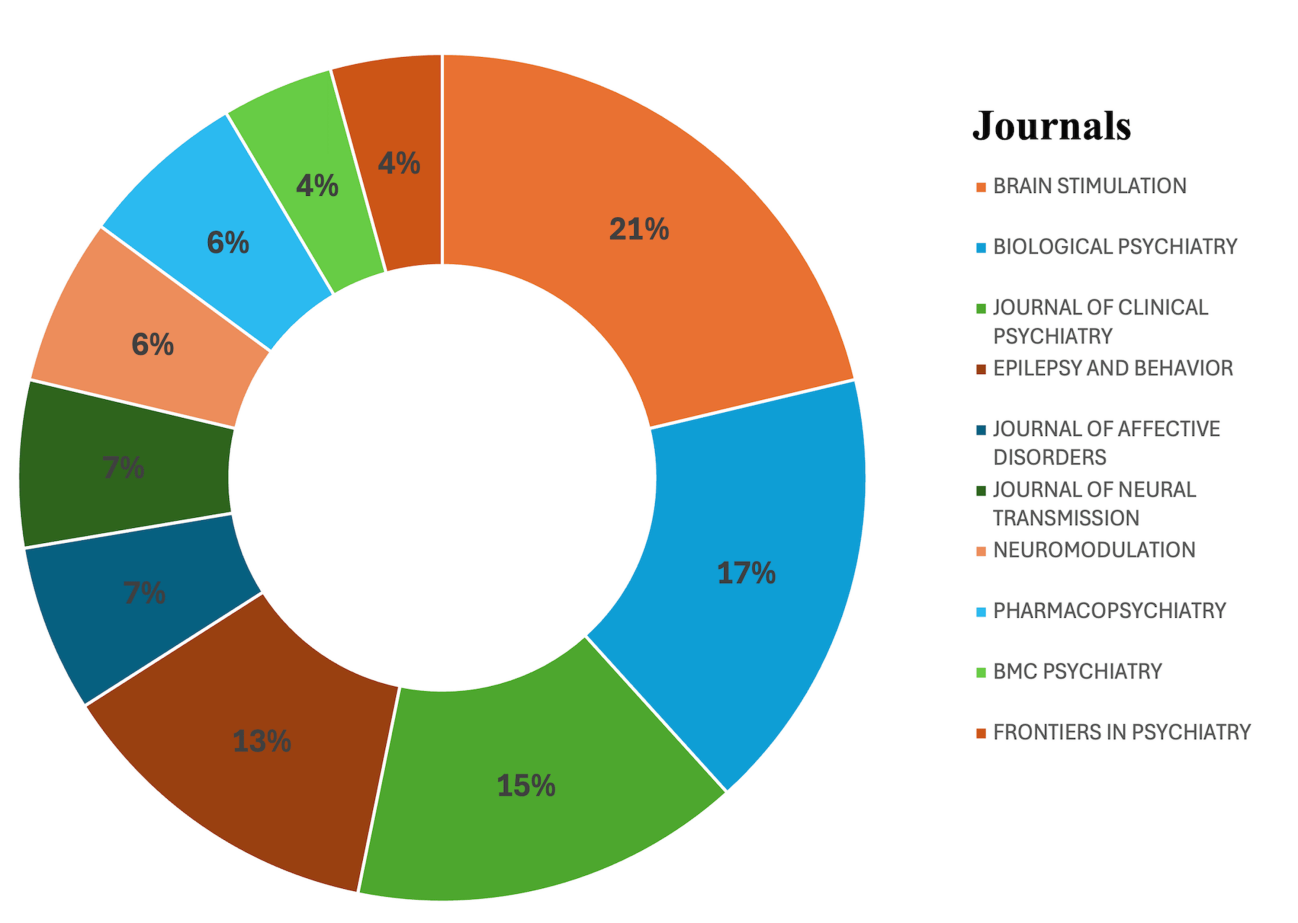
Distribution of articles among journals relating to VNS in psychiatric disorders VNS: vagus nerve stimulation This figure has been created by the authors using Microsoft Excel (Microsoft Corporation, Redmond, WA, USA).

The top-cited studies were published across 35 journals. Brain Stimulation led in frequency (10 articles), followed by Biological Psychiatry (8) and The Journal of Clinical Psychiatry (7), reflecting strong alignment with clinical neuroscience and translational psychiatry. Other notable contributors include Epilepsy and Behavior, Journal of Affective Disorders, and Neuromodulation. In terms of impact, Biological Psychiatry amassed 2,347 total citations. The Journal of Clinical Psychiatry accumulated 1,135 citations (h-index/g-index: 7; m-index: 0.292). Brain Stimulation followed with 632 citations and the highest combined h-index and g-index (10), m-index: 0.556. Frontiers in Psychiatry demonstrated strong citation efficiency, collecting 153 citations from only two recent articles (m-index: 0.25). Figures 3-4 visually represent these findings, showing journal-wise citation metrics and proportional article contributions. It should be noted that all h-, g-, and m-index values reported herein are sample-restricted indicators, calculated solely within the top-100 cited article subset, and should not be interpreted as journal-wide bibliometric metrics.

Geographic distribution and citation impact of scientific output

**Figure 5:**
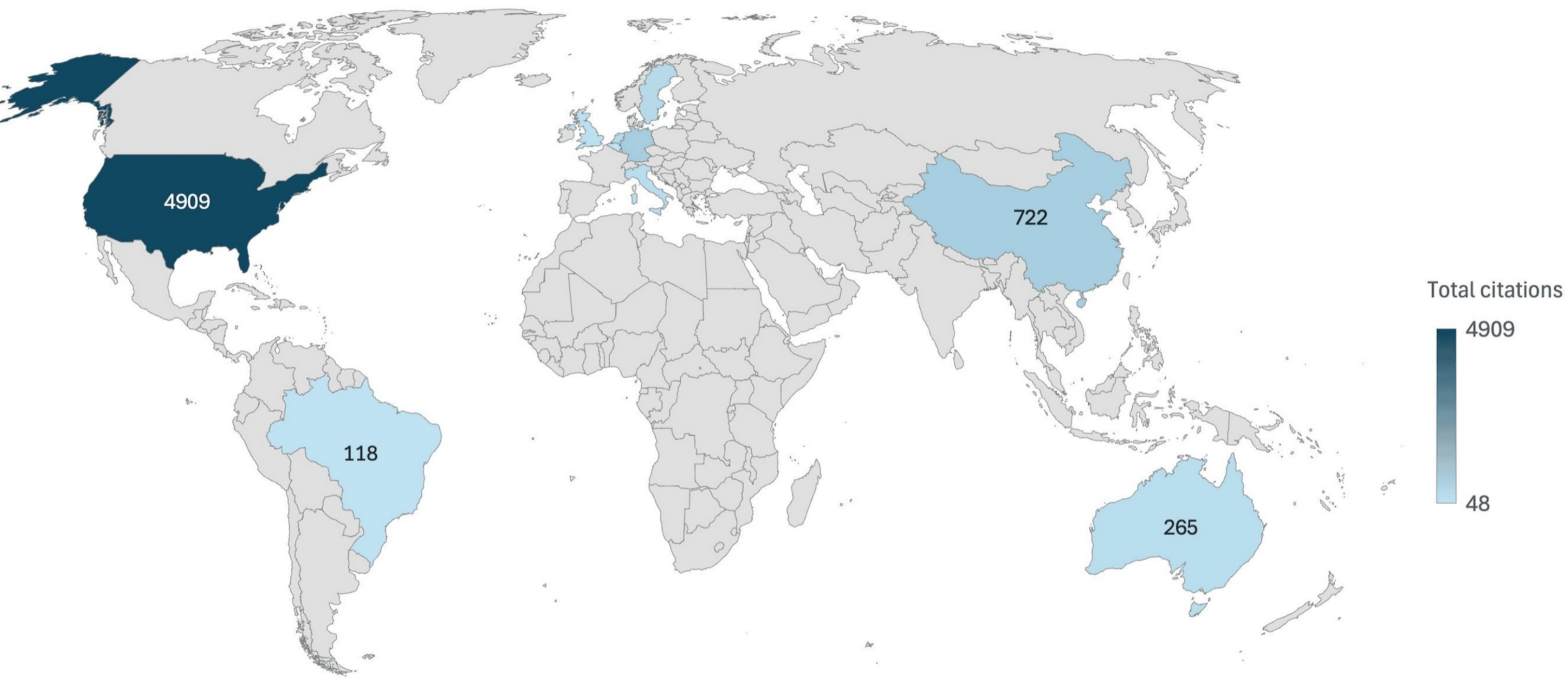
Global citation map of VNS research in psychiatric disorders VNS: vagus nerve stimulation The map in this figure has been created by the authors using Microsoft Excel (Microsoft Corporation, Redmond, WA, USA).

**Figure 6:**
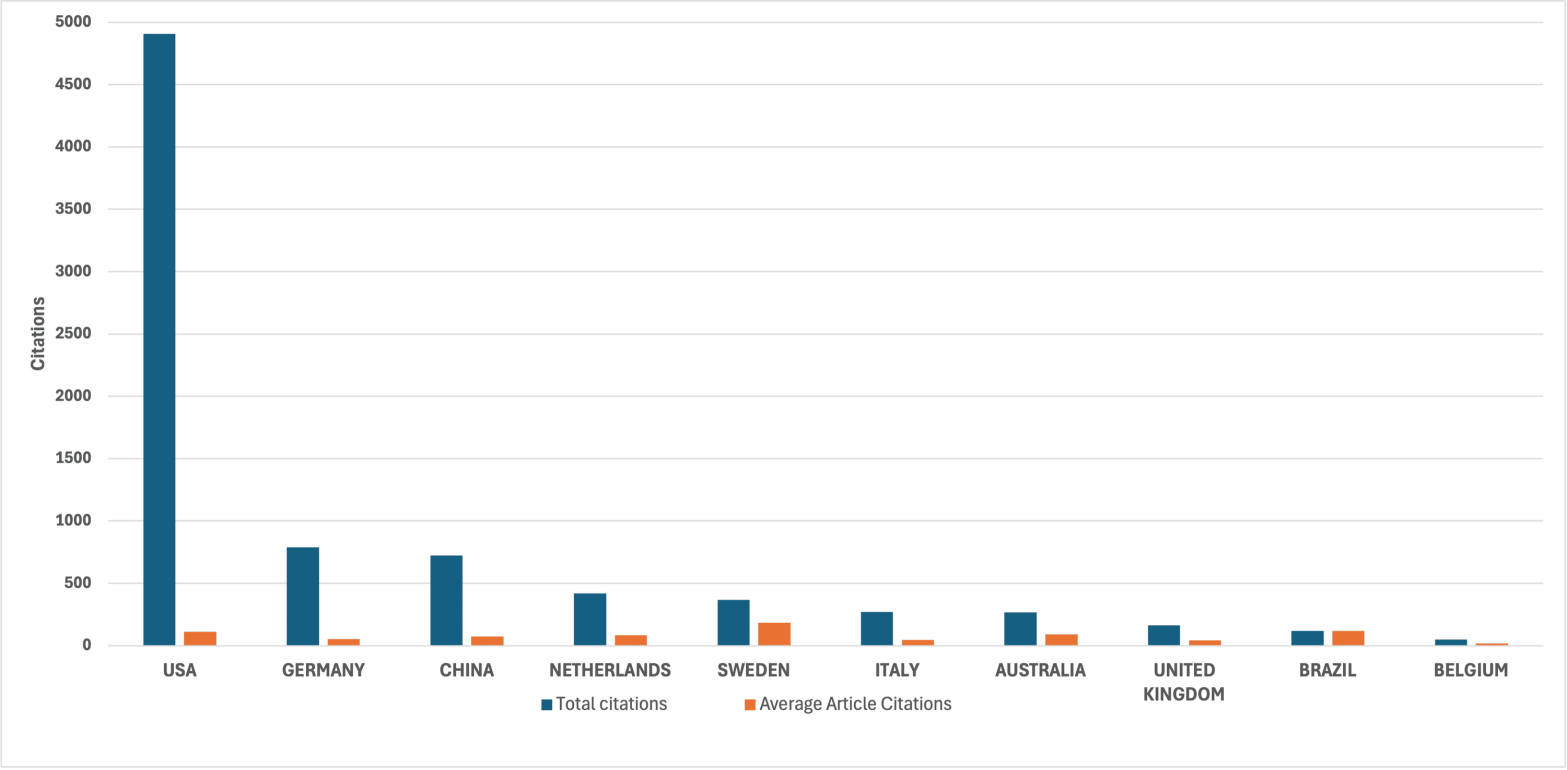
Citation impact among the most cited countries This figure has been created by the authors using Microsoft Excel (Microsoft Corporation, Redmond, WA, USA).

Among the author affiliations recorded across the top 100 cited articles, the United States accounted for 426 affiliation-level occurrences, reflecting both the volume of US-based co-authors and their dominant institutional representation, with an average of 111.6 citations per article among US-affiliated studies. China accounted for 114 affiliation-level occurrences, reflecting substantial research participation, although with a lower average citation impact per article. Sweden and the Netherlands, despite fewer affiliation-level occurrences, demonstrated comparatively strong citation efficiency. Additional contributions from Brazil and Australia further underscore the global diversification of influential research. Figures 5-6 present global citation maps and country-level contributions.

Institutional contributions

**Figure 7:**
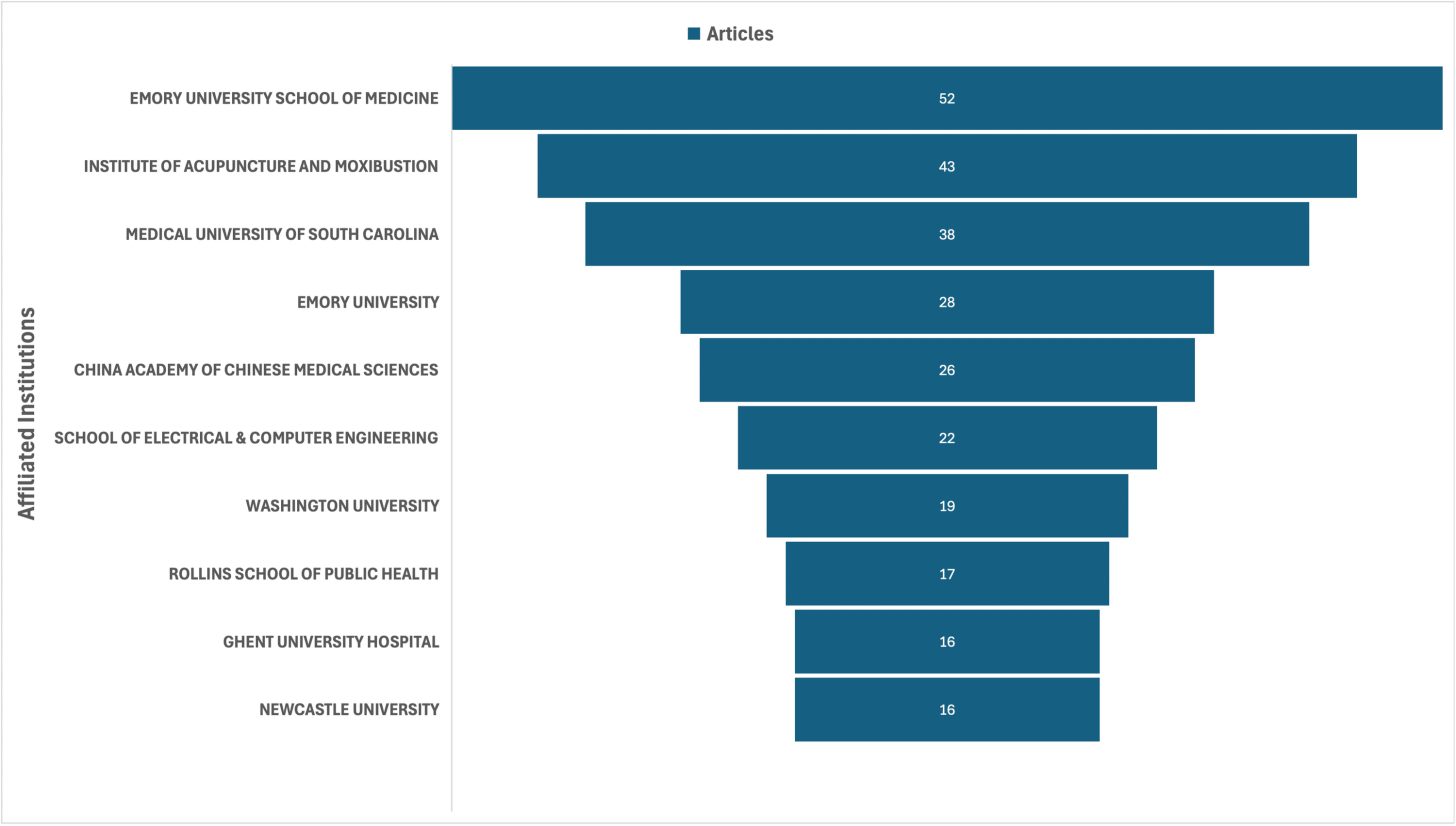
Institutional contributions of VNS research in psychiatric disorders VNS: vagus nerve stimulation This figure has been created by the authors using Microsoft Excel (Microsoft Corporation, Redmond, WA, USA).

The institutional distribution of VNS research shows a strong concentration among a few high-output centers. Emory University School of Medicine leads with 52 articles, followed by the Institute of Acupuncture and Moxibustion (43) and the Medical University of South Carolina (38), reflecting a central role in clinical and translational neuromodulation. The China Academy of Chinese Medical Sciences (26) and the School of Electrical and Computer Engineering (22) underscore growing Eastern contributions and clinical-technological leadership, respectively. Institutions such as Washington University, Rollins School of Public Health, Ghent University Hospital, and Newcastle University also contributed notably. As shown in Figure 7, a small number of institutions dominate output, emphasizing the importance of interdisciplinary capacity and infrastructure in advancing VNS research.

Keyword frequency in VNS research in psychiatry

Author keywords revealed dominant thematic clusters. The term "vagus nerve stimulation" and its variants appeared in more than 40 articles. Mood disorders dominated the clinical landscape, with "depression" cited in 19 articles, followed by "treatment-resistant depression" (11) and "major depressive disorder" (9), reflecting a clear emphasis on affective disorders. "Efficacy" and "side effects" appeared regularly, reflecting focus on treatment outcomes. The term "epilepsy" was also present, acknowledging VNS's therapeutic origins.

Discussion

This bibliometric analysis provides an in-depth examination of the evolution, current state, and future directions of VNS research in psychiatric disorders. By analyzing the 100 most cited original research articles published between 2000 and 2025, this study sheds light on the intellectual, institutional, and geographical landscapes that have shaped this field over the past two and a half decades.

Steady Growth and Emerging Interest in VNS

The 6.21% annual growth in VNS-related publications reflects psychiatry's expanding engagement with non-invasive neuromodulation. This trend is fueled by the clinical burden of treatment-resistant depression, which affects approximately one-third of patients, and by growing concerns regarding polypharmacy, adverse effects, and limited long-term efficacy of conventional psychotropics [13-15]. As psychiatry increasingly embraces circuit-based models of mental illness, interest in targeted interventions such as VNS has grown significantly [16]. This paradigm shift reflects not only clinical urgency but also educational opportunity, as psychiatry training programs are now tasked with introducing neuromodulatory approaches, including VNS, as part of the standard teaching curriculum. As the field evolves toward device-based and non-invasive interventions, residents and early-career psychiatrists must acquire a working understanding of VNS indications, side-effect profiles, and neurobiological mechanisms [17]. The COVID-19 pandemic further accelerated interest in non-invasive, home-based interventions [18]. Compared to invasive procedures like DBS, VNS remains more accessible and better tolerated, which increases its potential for broader clinical adoption and educational incorporation [19]. Transcutaneous VNS devices, designed for portability and self-administration, align with psychiatry's transition toward hybrid care delivery and may facilitate neuromodulation access in outpatient and primary care settings.

Temporal Peaks and Influencing Factors

While VNS publications steadily increased, distinct peaks in 2005 and 2007 likely reflect initial translational studies that validated its safety and efficacy in psychiatry, laying the foundation for expanding clinical use in mood and anxiety disorders. The most significant surge occurred in 2020, coinciding with the COVID-19 pandemic, which saw increased psychiatric burden, rapid adoption of at-home interventions, and an expansion of remote trial infrastructure [20,21]. Older articles from 2000 to 2002 maintain high citation density, likely due to early field dominance. However, recent studies from 2019 onward are gaining citations rapidly, reflecting greater visibility through open-access publishing, broader global collaboration, and increased interest among early-career psychiatry researchers [22].

Authorship Concentration and Journal Dissemination

The prominence of key figures such as A.J. Rush, M.S. George, and H.A. Sackeim in the most cited VNS research reflects not only scientific leadership but also the outsized influence of a few academic institutions on shaping the field's trajectory. While this continuity fosters methodological rigor, it also raises questions about equitable authorship opportunities, research gatekeeping, and the inclusion of early-career investigators in high-impact publications. For training programs, this pattern highlights the need to embed structured mentorship in scholarly activity. Integrating early exposure to publication pathways, bibliometric methods, and neuromodulation research into psychiatry residency curricula may address these access gaps [23].

Equally important is the evolving landscape of publication venues. While highly cited articles concentrate in neuroscience-heavy outlets like Biological Psychiatry or Brain Stimulation, platforms such as Frontiers in Psychiatry and Neuromodulation are gaining ground. These journals emphasize open access, interdisciplinary collaboration, and quantitative outcome reporting - features that align with the Accreditation Council for Graduate Medical Education's emphasis on evidence-based training and scholarly dissemination.

Institutional and Geographic Leadership in VNS Research

The United States continues to lead VNS-related psychiatric research in both publication volume and citation impact, reflecting its well-resourced research infrastructure, sustained neuromodulation funding, and early adoption of device-based psychiatric treatments, including VNS, TMS, and DBS [24]. Countries like Sweden, the Netherlands, and Brazil show high citation rates despite lower publication volumes, underscoring the impact of rigorous research from diverse regions. In contrast, developing countries remain underrepresented in VNS research, likely due to limited access to advanced technology, mental health stigma, lack of awareness, and insufficient funding [25]. This concentration of research within high-income settings risks limiting cross-cultural generalizability and reducing opportunities to examine population-specific moderators of treatment response [26]. Academic centers that integrate psychiatry residents into translational neuroscience labs or device innovation programs may better prepare them for future roles in precision psychiatry [27].

Clinical Applications and Expanding Horizons

The dominance of treatment-resistant depression in the VNS literature reflects a translational focus on disorders with high clinical burden. While this prioritization is understandable, it narrows the perceived relevance of VNS and limits opportunities for advancing its application across the diagnostic spectrum. VNS research linking stimulation to neural networks involved in emotion regulation, autonomic tone, and cognitive function can serve as an entry point for teaching circuit-level models of psychopathology [28,29]. Disorders such as PTSD, generalized anxiety, and somatic symptom presentations have shown preliminary responsiveness to vagal nerve modulation, yet remain underrepresented in top-cited psychiatric literature [19,30]. The future of VNS lies in expanding its empirical foundation across psychiatric diagnoses, investigating its neuroimmune and neurovisceral mechanisms, and evaluating patient-centered outcomes in real-world settings.

Limitations

Despite its comprehensive scope, this bibliometric analysis has notable limitations. The reliance on Scopus alone may have excluded relevant studies indexed in other databases such as Web of Science, PubMed, or Google Scholar, especially non-English or region-specific publications, thereby limiting global representativeness. Additionally, the top-100 citation-based design carries predictable structural biases: older articles benefit from longer citation accumulation windows; citation counts may be inflated by self-citation and dense collaborative networks; and high-income countries and flagship journals are disproportionately represented due to resource and indexing advantages. Citations per year were calculated to partially mitigate temporal skew. It should be explicitly noted that citation frequency reflects academic visibility and influence, not necessarily methodological quality or clinical validity. Keyword-based thematic analysis was dependent on author-assigned terms, which may vary in precision and consistency. The study did not include a formal assessment of methodological rigor or outcome measures, and no narrative synthesis of study content was performed. Finally, the limited representation of authors from low- and middle-income countries underscores ongoing disparities in research funding and signals a broader need for equity in psychiatric research visibility.

## Conclusions

This scientometric analysis offers a meta-academic lens into how research productivity, institutional affiliation, and thematic concentration have shaped the VNS psychiatric literature. For academic departments aiming to expand their footprint in neuromodulation, the results provide benchmarking data for author and institutional productivity, insight into citation dynamics that shape research influence, identification of high-yield journals and emerging subdomains such as PTSD and anxiety, and guidance on structuring mentorship pipelines in translational psychiatry. As neuromodulatory approaches enter mainstream psychiatric training, the insights from this study can directly inform curriculum development, grant strategy, and interdisciplinary collaborations at academic institutions.
